# A Case Report of an Adenomatoid Tumor of the Fallopian Tube: The Histopathologic Challenges and a Review of the Literature

**DOI:** 10.3390/jcm14030813

**Published:** 2025-01-26

**Authors:** Marcin Jozwik, Katarzyna Bednarczuk, Zofia Osierda, Joanna Wojtkiewicz, Janusz Kocik, Maciej Jozwik

**Affiliations:** 1 Department of Gynecology and Obstetrics, Collegium Medicum, University of Warmia and Mazury, 10-045 Olsztyn, Poland; 2 Scientific Circle of the Department of Gynecology and Obstetrics, 10-718 Olsztyn, Poland; 3 Department of Human Physiology and Pathophysiology, School of Medicine, University of Warmia and Mazury, 10-082 Olsztyn, Poland; 4 Clinical Hospital of Ministry of Interior with Warmia-Mazury Cancer Center, 10-718 Olsztyn, Poland; 5 School of Public Health, Center of Medical Postgraduate Education, 00-416 Warsaw, Poland; 6 Department of Gynecology and Gynecologic Oncology, Medical University of Białystok, 15-276 Białystok, Poland; maciej.jozwik@umb.edu.pl

**Keywords:** adenomatoid tumor, benign neoplasms, differential diagnosis, fallopian tube

## Abstract

**Background**: Adenomatoid tumor (AT) is a rare benign neoplasm of mesothelial origin, which mainly occurs in the male and female genital tracts. The most common site for AT occurrence in women is the uterus, which makes the presentation in the fallopian tube(s) a rarity with an incidence of approximately 0.5%. The reported extragenital sites include serosal surfaces, adrenal glands, and visceral organs, are even less common. Macroscopically, ATs present as white-grayish or yellowish irregular yet circumscribed firm nodules, often containing cystic components. Owing to a vast array of histomorphological growth patterns, ATs tend to mimic malignancy and trigger overresection. Such clinical situations have been described by several studies for the ovaries, uterus, and fallopian tubes, underlining the importance of differential diagnosis in order to avoid superfluous treatment. **Methods**: Herein, we report a presentation of an AT at the oviductal lumen, detected incidentally during prophylactic bilateral salpingo-oophorectomy in a 67-year-old patient with a BRCA1 mutation. **Results**: Immunohistochemical staining revealed a positive expression for calretinin, WT1, and cytokeratin 7, and negative expression for both PAX8 and CD34, thus confirming the diagnosis of AT and excluding tubal malignancy. **Conclusions**: This report, with a concise review of the global literature on tubal AT, brings attention to the solitary and asymptomatic nature of the tumor. With a clear diagnosis, no surgical radicality is necessary.

## 1. Introduction

Adenomatoid tumor (AT) is a rare benign neoplasm of mesothelial origin [1]. It was first tagged as “adenomyoma” in 1916 [2] and was renamed as “adenomatoid tumor” by Golden and Ash in 1945 [3], a designation widely accepted ever since. Typically, these tumors occur in the male genital system; however, their localization in the female genital tract is also possible, in which the most common site of occurrence is the uterus. Yet, ATs can be found in the fallopian tubes and ovaries. The tubal presentation is a rarity with an approximate incidence of 0.5% of all ATs in women. The reported extragenital sites are even less common and include serosal surfaces and adrenal glands as well as visceral organs [1].

For decades, ATs have been both a matter of controversy and a topic of multiple studies assessing their either mesothelial or endothelial origin. Nowadays, the most accepted viewpoint is that they represent benign mesothelioma [4,5,6,7,8]. Interestingly, for the oviductal localization, they were considered to arise from Müllerian vestiges [9]. At the cellular level, the tumor is composed of small glandlike spaces lined by flattened or cuboidal mesothelium-like cells. At the tissue level, it can present as many distinct cellular arrangements [1]. Therefore, owing to this vast array of histomorphological patterns, ATs can mimic malignant growths. Such situations have been reported for the uterus, ovary, and fallopian tubes [10,11,12,13]. Further, lesions of substantial sizes (such as of 10, 11.5, 12 and 13 cm diameter, or even over 20 cm in maximal dimension) [14,15,16,17,18] and of increased mitotic index [19,20] have all been described. The tumor’s tissue spread can be multifocal and diffuse infiltrative [19,21,22]. The lesion can simultaneously affect many organs [11,17]. Miller and Lieberman described three cases of AT with local invasion, albeit not expanding any further [23]. A uterine AT with regional lymph node involvement has been reported [24]. Another uterine AT with multi-cystic nodular lesions mimicked peritoneal carcinomatosis in a 66-year-old woman [11]. Finally, in an excellent molecular biology study from China, a concordant nonrandom X-chromosome inactivation pattern consistent with monoclonality was found, demonstrating that ATs represent a monoclonal disease favoring a neoplastic process [25]. Thus, care providers’ vigilance is justified. Such situations underline the importance of a careful differential diagnosis of AT in order to avoid the overresection of the given organ. The detection of ovarian and tubal ATs is mostly occasional at surgical excision and must be confirmed by immunohistochemistry (IHC) which is specific to the tumor.

In this article, we review the literature on AT in the female genital tract, with a particular focus on tubal AT, and on the differential of this tumor using histology and IHC staining. Our appraisal is substantiated by a case study of such a growth incidentally detected in a *BRCA1* mutation-carrier during a prophylactic bilateral salpingo-oophorectomy.

The presentation was written in line with the updated 2023 SCARE guidelines for surgical case reports [26].

## 2. Case Study

The patient was a 67-year-old Caucasian woman with familial ovarian cancer aggregation syndrome who presented to the gynecologic outpatient department shortly after she tested positive for a *BRCA1* mutation known to be a founding mutation for the Polish population. Her last menstrual period was at the age of 52 years. She opted for prophylactic and minimally invasive surgery. Therefore, a laparoscopic approach was chosen. The patient was normosthenic, in good general health and her preoperative laboratory workup, bimanual pelvic exam, and transvaginal ultrasound exam were all unremarkable. Specifically, except for an atrophied uterine corpus and a thin postmenopausal endometrium, no suspicious images of her ovaries and tubes were detected with a transvaginal ultrasound.

At surgery under general anesthesia, a somewhat atrophied uterus relevant to the patient’s age was confirmed, and the ovaries and Fallopian tubes seemed unsuspicious. Following the uncomplicated removal of both adnexa, the specimens were preserved in a fixative (10% neutral buffered formalin) and subjected to anatomopathological examination.

**Figure 1:**
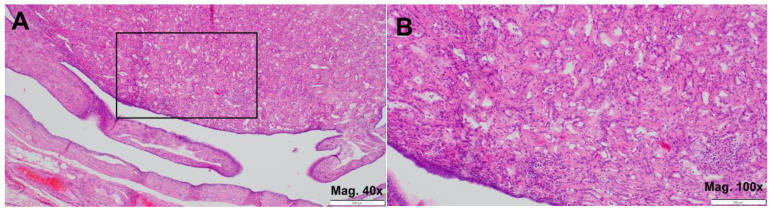
Adenomatoid tumor (H&E). (**A**) upper part-tumor tissue, lower part-tubal mucosa with signs of atrophy (magnification 40×); (**B**) details of magnified areal marked on (**A**) with visualized gland-like spaces (magnification 100×).

At gross dissection, a 0.5 cm cream-colored, not so well-circumscribed solid tumor was found in the distal part of the right fallopian tube. Microscopically, under hematoxylin and eosin (H&E) staining, it was found to be composed mostly of pseudoglandular formations (Figure 1B).

Further, tissue sections were stained for a wide array of specific antibodies (from Roche, Munich, Germany, and Sigma-Aldrich, Burlington, MA, USA) on Ventana Benchmark Ultra System (Roche Diagnostics) to identify the immunohistochemical (IHC) profile of the lesion. Immunohistochemistry is a crucial tool in distinguishing between similar-appearing lesions by identifying specific protein markers expressed in the tissue. For this study, the selection of calretinin, WT-1, cytokeratin 7, PAX8, and CD34 was based on their established roles in differential diagnosis of adenomatoid tumors (AT) and other potential mimics.

Calretinin and WT-1 are mesothelial markers which are highly sensitive and specific to mesothelial-derived lesions, making them essential in confirming the mesothelial origin of AT. Cytokeratin 7, a broad epithelial marker, is often expressed in mesothelial and epithelial tumors, helping to differentiate AT from non-epithelial lesions. PAX8, a marker commonly expressed in epithelial neoplasms of Müllerian or renal origin, is typically absent in mesothelial lesions like AT, serving as a negative control. Similarly, CD34, an endothelial marker, helps exclude vascular tumors that may mimic AT morphologically.

**Figure 2:**
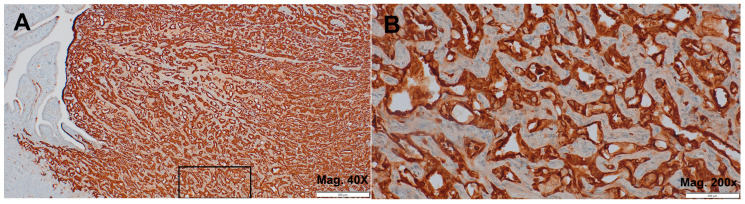
Adenomatoid tumor. (**A**) a highly positive IHC staining for calretinin (magnification 40×); (**B**) details of magnified areal marked on (**A**) (magnification 200×).

**Figure 3:**
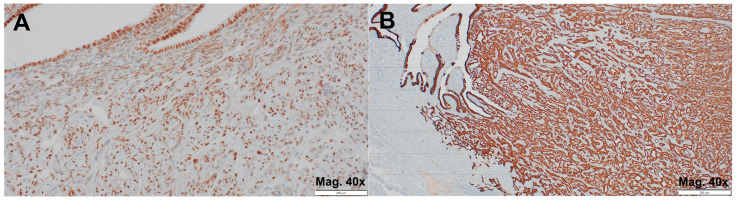
Adenomatoid tumor. (**A**) a positive IHC staining for WT-1 (magnification 40×); (**B**) a highly positive IHC staining for cytokeratin 7 (magnification 40×).

With positive IHC expression for calretinin (Figure 2A,B), WT-1 (Figure 3A), and cytokeratin 7 (Figure 3B), along with negative expression for both PAX8 and CD34 (not shown), the immunoprofile was consistent with AT, thereby confirming the diagnosis.

## 3. Discussion

In clinical terms, even if rare, ATs represent the most common benign tumor of the fallopian tube [27], in its wall and mesosalpinx combined [28]. There are no hard data on the factual AT incidence in the female genital tract, albeit fallopian tube ATs are commonly believed to be rarer than their uterine equivalent. The relative incidence of uterine ATs was given in several papers. An early prospective study revealed that 1 AT in 100 consecutive hysterectomy specimens examined had a frequency of 1.0% [29]. In the Tiltman study, 1000 hysterectomy specimens were histologically examined, and 14 ATs were found in 12 uteri, at frequency of 1.2% [30]. In 2016, Mizutani et al. reported that during the 8-year study period of 1094 total hysterectomies 17 (1.55%), such lesions were found [31]. Yet, in an earlier Japanese work, the authors observed the findings from 199 consecutive gynecologic surgeries: ATs were identified in 6 (4.5%) out of the 134 hysterectomy specimens, and 3 (4.6%) out of the 65 uterus-preserving tumor excisions [32]. Because all these data relied on preselected morbid populations, we roughly and conservatively estimate that the relative incidence of fallopian tube ATs in total hysterectomy specimens is less than 1.55%. Last year’s account of fallopian tube AT reported 49 published cases and presented 1 new case [33]. Thus, our case study would be the report on the fifty-first patient. One possible underestimation stems from the study by Jablokow et al., where all the female genital tract cases (*n* = 4) were tubal [28].

In 2001, Nogales et al. classified uterine ATs based on the presence of distinct macroscopic patterns into two types: 1. small (up to 3.5 cm) solid tumors, and 2. large cystic tumors [19]. This classification may apply to other gynecologic sites of AT. We failed to detect any preferred location of AT’s occurrence within the fallopian tube. It is most frequently a unilateral lesion. A mention of the presence of ATs in both tubes of a patient was stated by Youngs and Taylor [34]. Due to the asymptomatic nature of the tumor, no characteristic signs and symptoms have been outlined. This seems particularly true for the oviductal lesions, whose size was generally reported as smaller than in the case of uterine lesions [21,34]. We think, however, that some clinical symptoms can be related to the lesion’s location. For instance, menorrhagia for the past few months was the first manifestation of a uterine AT [10]. In the Srigley and Colgan study, the eccentric mural nodule’s growth entirely distorted the tubal lumen [21]. A similar tubal displacement and compression was presented in an earlier work [34]. Two studies related tubal ectopic pregnancy to the tubal obstruction from AT [27,35]. In the uterine location, there is a rare possibility of detecting signet-ring cells in curettings, for instance during investigation for infertility [36] or due to heavy menstrual bleeding [10]. The reason for such a finding is that uterine ATs contain signet-ring cells more often than ATs in other gynecologic locations, which is perhaps an organ-related peculiarity. In the Ersan Erdem study, none of the 4 tubo-ovarian cases revealed any signet-ring cell formation, whereas these cells were present in 6 of 10 uterine tumors [37].

ATs can be assigned to a plethora of specific histologic patterns: angiomatoid, adenoid, solid, cystic, glandular, oncocytic and tubular. Many cases reveal a combination of different patterns present in one tumor [1,37,38]. The angiomatoid pattern consists of flat neoplastic cells resembling endothelial cells. Wide, pseudovascular channels are separated from each other by smooth muscle cells bundled into fascicles. The presence of aggregated lymphocytes may give a false impression of a lymphangioma [39]. The adenoid pattern is characterized by fibrous tissue with anastomosing gland-like structures edged by mesothelial-like cuboid cells and the majority of neoplastic cells being vacuolated [39]. In the solid pattern, the neoplastic cells are grouped in longitudinal systems, have an eosinophilic cytoplasm and do not contain as many vacuoles as seen in the adenoid pattern. The cystic pattern consists of many cystic cavities separated by thin cords of neoplastic cells accompanied by connective tissue. Tumors with this pattern tend to be well-circumscribed [39]. Pseudolipoblasts and signet-ring cells may be present in the microscopic field as well [21,40]. The fibrous connective tissue can be a substantial part of the tumor, especially in the solid and adenoid patterns. Both these patterns must be clearly distinguished from metastatic carcinomas [40]. Elastin content in ATs is varied [41]. Electron microscopic studies demonstrated, among others, the abundance of microvilli on the surface of the tumor cells [21,41,42], an argument for the mesothelial origin of ATs. Although many lesions display more than one pattern of the morphologic arrangement, a uniting feature, thread-like bridging strands are present in all ATs [43]. The above summarized architecture of ATs is relevant for both the final postoperative anatomopathological examination and extemporaneous intraoperative verification.

As to IHC, ATs in both the female and male genital tracts react strongly with pan-keratin (membranous and cytoplasmic), calretinin (nuclear and cytoplasmic), D2-40 (nuclear and/or cytoplasmic), and WT-1 (nuclear) [13,38,44,45]. The membranous and cytoplasmic reactivity with pan-keratin is the most significant [37,38]. In the presented case, stainings for calretinin and WT-1 were both strongly positive. The first compound is a calcium binding protein. WT-1 is the product of the expression of the Wilms’ tumor suppressor gene [45]. Pan-keratin antibodies are mixtures of two or several antibodies that detect multiple low- and high-molecular-weight keratins. In our case, an antibody to cytokeratin 7 was used instead and revealed a positive reaction, as described [37]. In line with the literature [38], vimentin, PAX8, and CD34 remained negative in our stainings. At that point, D2-40 (a monoclonal antibody that reacts to podoplanin, a small transmembrane O-linked sialoglycoprotein) staining was not crucial for the diagnosis and, therefore, was not performed. All tumors were negative to CK-20, CD10, CD31, and CD68 [37]. A particularly broad array of IHC antibodies were tested on ATs by Terada [46]. A thorough discussion of the topic was conducted by Karpathiou et al. [47].

Notably, somatic missense mutations in *TRAF7* (tumor necrosis factor receptor-associated factor 7 gene) were observed in ATs, with most common alterations being p.H521R, p.S561R, and p.Y538S [12,48]. *TRAF7* encodes an E3 ubiquitin protein ligase and, through that, reduces the transcriptional activity of the nuclear factor-κB (NF-κB) protein complex. *TRAF7* mutations cause the activation of the NF-κB signaling pathway that may be behind the link between ATs and autoimmune diseases or immune dysregulation [12,48]. Examples of this association include patients with chronic renal failure, with renal transplant and immunosuppressed [31,49,50,51], and with chronic hepatitis C virus infection [52]. An impressive study by Tamura et al. analyzed 611 consecutive hysterectomy specimens to determine the incidence of AT and its correlation with the immunosuppressive status [53]. Overall, ATs were detected in 14 hysterectomy cases (2.3%), yet this incidence was significantly higher in the immunosuppressed patients (25.0%) than in the non-immunosuppressed patients (1.52%), a relative risk of 16.45. Observations on ATs of extragenital location provide additional evidence for the link [54,55]. Further research is warranted in this area. Our patient was not diagnosed with TRAF7 gene mutation, however, she was a proven carrier of a *BRCA1* mutation.

The microscopic and IHC diagnosis of AT can be simple and straightforward, or not necessarily so. Similarly, the differential can be difficult. Clinical, gross, and microscopic features of the tumor are obviously not sufficient [37] and the implementation of other techniques, such as histochemistry or fluorescence in situ hybridization (or, FISH) need to be taken into consideration for correct diagnosis. The concurrent goals are to confirm the diagnosis of AT and exclude the presence of any malignancy. Preparations containing extensive necrosis, presumably due to infarction, may be diagnostically challenging [56].

The differential diagnosis must include malignant mesothelioma. Originating from the mesothelial lineage, it gives similar IHC reactions as AT, such as positivity for pan-keratin, cytokeratin 7, cytokeratin 5/6, CR, WT-1, D2-40, and a negative reaction to cytokeratin 20, CD-10, CD-31, and vimentin. Possible patterns of malignant mesothelioma are tubular, papillary, and solid, as it mostly presents as a large infiltrative mass with mild to moderate cytologic atypia and mitotic activity [13,37]. For the diagnosis of a particular subset of the tumor, malignant mesothelioma with prominent adenomatoid features, Weissferdt et al. recommended a panel including CEA, CD15, MOC31, B72.3, broad-spectrum keratin AE1/AE3, cytokeratin 5/6, calretinin, and TTF1. In all their cases, the tumor cells showed a strong positive reaction for broad-spectrum keratin, cytokeratin 5/6, and calretinin, whereas all the other markers were negative [57]. Adenocarcinoma can mimic ATs by its trabecular or trabecular/microcystic pattern. Microscopically, the cells demonstrate cytologic atypia and possess the ability to infiltrate. The absence of mesothelial markers expression differentiates it in IHC from AT [13,37]. Lymphangiomas can resemble cystic spaces of ATs with their cystically dilated lymphatic spaces, although they contain lymphocytes and therefore are negative for calretinin. On the other hand, they can test positive for D2-40, as well as ATs [13]. Well-differentiated papillary mesothelioma is a polypoid peritoneal lesion with one of many different patterns including papillary, tubulopapillary, adenomatoid-like, and branching cords. In IHC, it reacts positively to CK, CR, cytokeratin 5/6, WT-1, D2-40, L1CAM making it similar to AT [37].

In reference to the differential diagnosis, Weiss and Tavassoli suggested an interrelationship between epithelial mesothelioma, cystic mesothelioma, and AT [58]. And De Rosa et al. believed that cystic giant AT of the uterus bridges the gap between typical AT and cystic mesothelioma because it has features of both these lesions [16]. As mentioned, ATs are genetically defined by frequent harboring *TRAF7* mutations. In a recent study comparing uterine ATs with pleural or peritoneal malignant mesotheliomas, somatic mutations of *TRAF7* were confirmed by next-generation sequencing in 84% (37/44) of the studied ATs and in none (0/21) of mesotheliomas. Thus, *TRAF7* alterations seem to be one of the major factors distinguishing both entities [59].

Looking at reports with the largest numbers of observations on AT in search of its common features, we could only point out the solitary and asymptomatic nature of the tumor [1,19,31,37,38,58,60,61].

Finally, our patient is not the first to carry a *BRCA1* mutation and have a tubal AT. In 2018, Lee et al. presented a similar case study of a 61-year-old woman with *BRCA1* mutation undergoing a prophylactic gynecologic procedure involving a bilateral salpingo-oophorectomy. The surgeon discovered a white nodule on the fimbriated end of the right fallopian tube and sent the right salpingo-oophorectomy specimen for intraoperative consultation [62]. Both cases, the Lee study and the current, support the notion that ATs develop with ease in patients with decreased immunity [48], and BRCA mutations may be the predisposing factor. Let us point out a recent report where BRCA1 promoter-hypermethylated ovarian cancers were characterized by the decreased composition of lymphocytes [63]. In the uveal melanoma microenvironment, BRCA1-associated protein 1 (or, BAP1) mutations were found to inhibit the NF-κB signaling pathway, suppressing the cytokine secretion and antigen presentation by macrophages [64].

## 4. Conclusions

Concisely, AT is mostly a rare incidental solitary finding in the female genital tract. Such tumors can usually be assigned to a specific histologic pattern; however, many lesions reveal more than one morphological arrangement. Since they can mimic malignancies, the differential diagnosis must be conclusive. The most relevant IHC stainings to confirm the diagnosis of AT include pan-keratin/cytokeratin 7, calretinin, WT-1, and D2-40 positivity, and PAX8, CD34, and vimentin negativity. The concurrent goal is to exclude the presence of any malignancy, including malignant mesothelioma and oviductal adenocarcinoma. With a clear diagnosis, no surgical radicality is necessary.

## Data Availability

The data presented in this study are stored in the hospital registry.
